# Valproic Acid Downregulates Cytokine Expression in Human Macrophages Infected with Dengue Virus

**DOI:** 10.3390/diseases6030059

**Published:** 2018-07-06

**Authors:** Félix G. Delgado, Paola Cárdenas, Jaime E. Castellanos

**Affiliations:** Grupo de Virología, Vicerrectoría de Investigaciones, Universidad El Bosque, Bogotá D.C. 110131, Colombia; fdelgadot@unbosque.edu.co (F.G.D.); paolaandrea192@hotmail.com (P.C.)

**Keywords:** dengue virus, macrophage, cytokines, HDAC inhibitors, valproic acid

## Abstract

Natural infection with dengue virus (DENV) induces an increase in the production of cytokines that play an important role in disease pathogenesis. Despite numerous scientific studies, there are still no commercially available disease-specific therapeutics. Previous evidence shows that inhibiting histone deacetylase enzymes (HDACs) regulates the immune response in several inflammatory disease models. The aim of the current study was to evaluate the effect of HDAC inhibition in the production of inflammatory cytokines in human monocyte-derived macrophages infected with DENV serotype 2 (DENV-2). To this end, human monocyte-derived macrophages (MDMs) were treated with valproic acid (VPA) before or after infection and the inflammatory cytokine concentration was quantified by flow cytometry. We found that infected MDMs secreted IL-8, IL-1b, IL-6, TNF-alpha, and IL-10, but not IL-12. Strikingly, treatment of infected cells with VPA had a differential and concentration-dependent effect on the production of specific cytokines without eliciting significant changes in cell viability. Using the highest concentration of VPA, a significant reduction in the production of all cytokines was observed. These results suggest that HDAC inhibition during DENV-2 infection could exert an important regulatory effect in the production of inflammatory cytokines, representing a significant advance in the design of novel therapeutic dengue treatments.

## 1. Introduction

Infection with dengue virus (DENV) presents a wide range of clinical symptoms and is the most important arboviral disease worldwide in terms of morbidity, mortality, and economic impact [1]. Most DENV infections remain asymptomatic with only a few progressing to mild symptoms, but the most severe forms of the disease are characterized by a rapid increase in inflammatory mediators, inducing endothelial activation and organ damage, which is associated with mortality [2,3].

This ‘cytokine storm’ is characterized by high levels of circulating cytokines and chemokines, which are produced mainly by T cells, monocytes, and macrophages. Increased levels of TNF-α, IL-1β, IL-4, IL-6, IL-8, IL-13, IL-15, macrophage migration inhibitory factor (MIF), and chemokines CCL2, CCL4, CCL5, and CXCL10 (IP-10) among others, have been reported in patients with dengue hemorrhagic fever (DHF) when compared to dengue fever (DF) [2,3].

Unfortunately, there is no a DENV-specific treatment, and the only available therapeutic is symptomatic and supportive management, although recent candidate vaccines have been tested with partial success [4,5]. Furthermore, there is no way to sustainably control mosquito populations in endemic areas. Thus, the search for new therapeutic agents for the control of DENV disease or the immunopathological features of severe disease is a major worldwide challenge.

It has been shown that inhibition of a group of enzymes with histone deacetylase activity modulates the immune response triggered by several pathogens and toll-like receptor (TLR) ligands in macrophages [6,7]. These histone deacetylase enzymes (HDACs) have an important cellular role in mediating different biochemical and epigenetic processes involved in gene expression by eliminating acetyl groups from histone or non-histone proteins [8]. It has been proposed that altering the acetylation balance of histone or non-histone proteins could underlie the sustained transcription of pro-inflammatory genes, thereby inducing a severe inflammatory process [9,10].

Valproic acid (VPA) is a well-known drug used for the treatment of epilepsy and bipolar disease. Its mechanism of action involves an increase in cerebral levels of gamma amino butyric acid (GABA), an inhibitory neurotransmitter [11]. However, it has been reported that VPA inhibits HDAC activity and has been proposed as candidate drug for cancer therapy due to its ability to induce differentiation in several types of transformed cells [8,12]. Furthermore, VPA and other HDAC inhibitors, such as trichostatin A (TSA) and vorinostat (suberoylanilide hydroxamic acid, SAHA), have been used in in vivo models of several inflammatory diseases to induce changes in the expression of inflammatory cytokines, demonstrating their immunomodulatory effect [8,13].

At present, the immunomodulatory effect of HDAC inhibitors has not been described during flaviviral infection. It is therefore unknown whether they are capable of controlling the secretion of inflammatory mediators during dengue infection. In the present study, we describe the effect of HDAC inhibition on the inflammatory response in human macrophages infected with DENV. Measurement of the concentration of inflammatory cytokines in cell supernatants allowed us to observe significant decreases in the level of IL-8, IL-1b, IL-6, TNF-alpha and IL-10 in cells treated with VPA before or after infection with DENV serotype 2, without affecting cell viability. Our results provide new insight that could guide the development of pharmacological treatments for dengue virus infection.

## 2. Materials and Methods

### 2.1. Dengue Virus

DENV serotype 2 (DENV-2; COL-789) was kindly donated by the Instituto Nacional de Salud de Colombia. The virus was harvested from C6/36 cells (ATCC^®^ CRL-1660™) at 6 days post-infection. The infected monolayer was subjected to three consecutive freeze/thaw cycles and the supernatant was centrifuged, aliquoted and stored at −80 °C until use. Viral titer (focus forming units/mL) was obtained by inoculating serial dilutions of viral stock on LLC-MK2 cell monolayers (ATCC^®^ CCL-7™), following the protocol reported previously [14].

### 2.2. Peripheral Blood Mononuclear Cell (PBMC) Purification

PBMCs were obtained from healthy donors who had previously authorized the use of blood samples. This study was approved by the Institutional Review Board (IRB) of Universidad El Bosque (Bogotá, Colombia). All sera had previously tested negative for dengue IgM and IgG antibodies using the Dengue Duo Cassette (Panbio Diagnostics, Windsor, Queensland, Australia). PBMCs were isolated by Ficoll density gradient centrifugation (Histopaque-1077, Sigma-Aldrich, St. Louis, MO, USA) and cultured in RPMI-1640 medium (Gibco, Life Technologies, Grand Island, NY, USA) supplemented with 10% FBS and antibiotics. PBMCs were grown using pyrogen-free certified reagents (endotoxin level <0.3 EU/mL) in a 37 °C, 5% CO_2_ humidified atmosphere.

### 2.3. In Vitro Generation of Monocyte-Derived Macrophages (MDMs)

Monocytes were obtained from PBMCs by positive selection using magnetic-activated cell sorting (MACS) beads and MACS separation columns (Miltenyi Biotech, Bergisch Gladbach, Germany) according to the manufacturer’s instruction. Monocyte purity (90–92%) was determined by analysis of CD14 expression (Figure S1A) using a FACSCanto™ II flow cytometer (Becton Dickinson, Erembodegem, Belgium) and FACSDiva™ software (Becton Dickinson, Erembodegem, Belgium). As previously shown [15], the monocytes were allowed to spontaneously differentiate into MDMs by seeding for seven days in RPMI 1640 medium (Gibco) supplemented with 10% human AB serum (Figure S1B,C).

### 2.4. Cell Viability Assay

After the treatment of the PBMCs with different concentrations of TSA or VPA for 3, 6 or 24 h in a 96-well plate, the medium was replaced with 100 μL of medium supplemented with 44 mM resazurin. The cells were incubated in triplicate for 4 h, and the fluorescence intensity was quantified using a spectrofluorometer (excitation wavelength: 535 nm; emission wavelength: 595 nm). The percentage viability was calculated by dividing the fluorescence reading of the cells under treatment by the fluorescence reading of the cells under normal growth without treatment.

### 2.5. Treatment with HDAC Inhibitors and Cytokine Quantification

PBMCs and MDMs were infected with DENV-2 using a multiplicity of infection (MOI) of 1. Depending on the assay, the cells were treated with TSA (100, 200 or 400 nM) or VPA (1, 2, or 4 mM) for three hours before or after infection. Cells inoculated with cell lysate from non-infected C6/36 cells were used as negative controls. Supernatants were collected at 24 h post-infection and used for cytokine detection by flow cytometry with a multiplexed fluorescent bead-based immunoassay (BD CBA Flex Set and BD CBA Human Inflammatory Cytokines Kit, Becton Dickinson) following the manufacturer’s instructions. We first used the CBA Flex Set, which included two cytokines with detection limits (expressed in pg/mL) as follows: interleukin 6 (IL-6), 1.6; tumor necrosis factor-alpha (TNF-alpha), 0.7. Subsequent experiments used the CBA Human Inflammatory Cytokines Kit, which probes for 6 cytokines with detection limits (expressed in pg/mL) as follows: IL-8, 3.6; IL1beta, 7.2; IL-6, 2.5; IL-10, 3.3; TNF-alfa, 3.7; and IL-12p70, 1.9. Data acquisition and analyses were performed using a FACSCanto™ II (Becton-Dickinson) flow cytometer equipped with FACSDiva™ and FCAP Array™ software (BD Biosciences, Erembodegem, Belgium).

### 2.6. Cytokine mRNA Quantification

Three hours post-infection, total RNA was isolated from PBMC cultures using Trizol Reagent (Invitrogen, Carlsbad, CA, USA) according to the manufacturer’s protocol and was solubilized in water treated with diethyl pyrocarbonate 0.1% (*v*/*v*). Isolated RNA was quantified and cDNA was synthesized from 0.5–1 µg of RNA per sample using random primers (Promega, Madison, WI, USA) and 250 U of MMLV (Promega). Relative expression was then quantified using the DyNAmo HS SYBR Green qPCR kit (Finnzymes, Espoo, Finland) in a real-time thermocycler (CFX96™ Real-Time System, Biorad, Hercules, CA, USA). The following primers were used: β-Actin forward 5′-GAT CAT TGC TCC TCC TGA GC-3′, β-Actin reverse 5′-ACT CCT GCT TGC TGA TCC AC-3′; TNF-alfa forward 5′-TGA AGG GAA TGG GTG TTC AT-3′, TNF-alfa reverse 5′-GAG TTG GAC CCT GAG CCA TA-3′; and IL-6 forward 5′-GAC AGC CAC TCA CCT CTT CA-3′, IL-6 reverse 5′-CCT CTT TGC TGC TTT CAC AC-3′. Relative expression of cytokine genes was calculated using a mathematical method previously described [16].

### 2.7. Apoptosis Assay

PBMCs were treated or not treated with VPA (1, 2, or 4 mM). At 24 h post-treatment, the cells were fluorescently labeled to detect apoptotic cells using phycoerythrin (PE)-conjugated Annexin V (Pharmingen, San Diego, CA, USA) and for monocyte identification using a fluorescein isothiocyanate (FITC)-labeled mouse anti-human CD14 (Pharmingen, San Diego, CA, USA) antibody. The samples were mixed gently and incubated at 4 °C in the dark for 30 min. After washing, a minimum of 10,000 events within the gated region (CD14+ cells) were analyzed using a FACSCanto™ II (Becton-Dickinson) flow cytometer and FACSDiva™ software (BD Biosciences).

### 2.8. Statistical Analysis

Statistical significance was determined using the non-parametric two-tailed Mann-Whitney U test to compare two independent groups. A *p* value < 0.05 was considered significant.

## 3. Results

### 3.1. HDAC Inhibition in PBMCs Infected with DENV-2 Induces a Decreased TNF-Alpha and IL-6 Production

**Figure 1:**
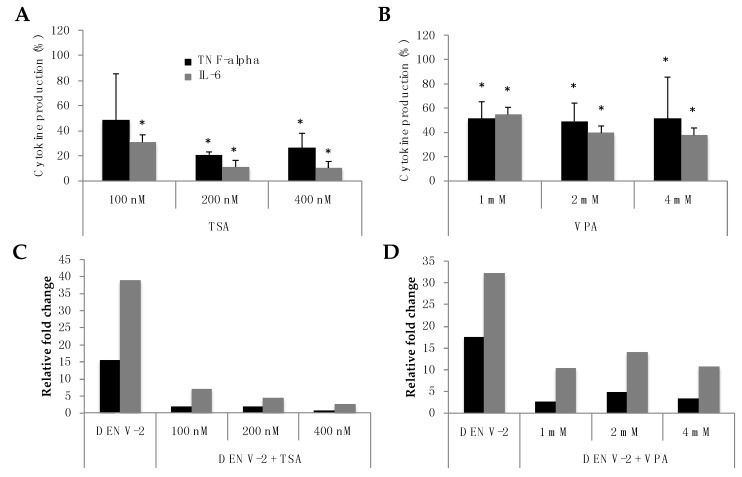
Trichostatin A (TSA) and valproic acid (VPA) pretreatment of peripheral blood mononuclear cells (PBMCs) infected with dengue virus (DENV-2) exhibit a decrease TNF-alpha and IL-6 production. PBMCs were treated with TSA (**A**) or VPA (**B**) for 3 h and then were infected with DENV-2. At 24 h post-infection, the supernatants were used to quantify cytokine production. Data is expressed as relative cytokine production compared with infected non-treated control cells. Error bars represent the mean ± SEM of three independent experiments. * *p* values were calculated using the non-parametric two-tailed Mann-Whitney test, comparing each experimental condition with the infected non-treated control. A *p* value < 0.05 was considered significant. PBMCs were treated with TSA (**C**) or VPA (**D**) for three hours and were then infected with DENV-2. At 3 h post-infection, total RNA was collected to quantify cytokine mRNA. Gene expression was normalized to human β-actin expression. The figure shown is representative data from one of three independent experiments.

We previously reported that PBMCs infected with DENV-2 produce and secrete TNF-alpha and IL-6 [17,18]. Using the same experimental culture approach, we found that TSA or VPA treatment induces a reduction in the secretion of both cytokines (Figure 1). When the pan-HDAC inhibitor TSA was delivered before infection, 76% and 73.4% reductions of TNF-alpha concentration in the supernatant were observed at 200 and 400 nM, respectively. Meanwhile, TSA treatment pre-infection induced a significant decrease of IL-6 production at all concentration tested ranging from 69% to 89.6% compared with infected non-treated control cells (Figure 1A).

Furthermore, after treatment with VPA (an HDAC I and IIa inhibitor), a significant reduction of TNF-alpha and IL-6 production was detected. However, the immunomodulatory effect exerted by VPA was less potent than that of TSA. As shown in Figure 1B, the mean reduction percentage of TNF-alfa production was detected between 48.5% and 50.7%. The percentage reduction in IL-6 expression ranged from 45.1% to 62.1%, and no significant difference was detected between the results obtained at different concentrations of VPA.

Transcripts for the genes TNF-alpha and IL-6 were quantified in DENV-2-infected PBMCs and in those treated with TSA or VPA. Infection induces significant cytokine mRNA over-expression (15- and 38-fold for TNF-alpha and IL-6 respectively), which was significantly down-regulated by the TSA treatment until basal non-infected cells level (Figure 1C). We observed the same effect on cytokine transcription, albeit at a smaller magnitude, in PBMCs treated with VPA. The addition of 4 mM VPA to culture medium before infection, significantly induced a 5- and 3-fold reduction in TNF-alpha and IL-6 mRNA expression, respectively (Figure 1D).

### 3.2. HDAC Inhibition Have Not a Significant Effect in Cell Viability

**Figure 2:**
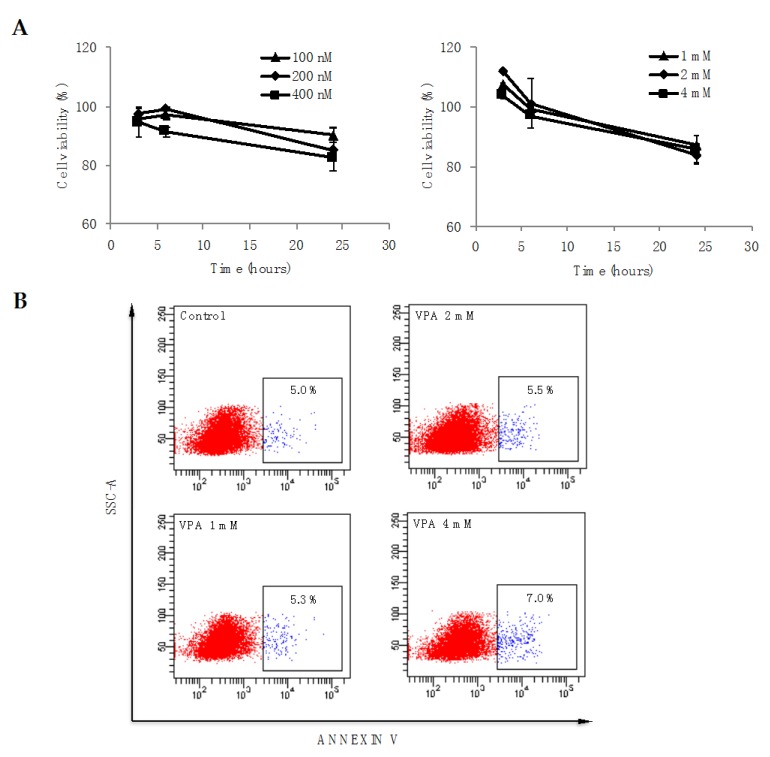
Cell viability of PBMCs pre-treated with TSA and VPA. (**A**) PBMCs were treated with different concentrations of TSA (left panel) or VPA (right panel) and their metabolic activity was quantified after 3, 6 and 24 h using resazurin. The percentage viability was calculated as described in the methods section. Error bars represent the mean ± SEM of three independent experiments; (**B**) PBMCs were treated with different concentrations of VPA, and after 24 h, CD14+ cells were analyzed for apoptosis using Annexin V.

To determine whether treatment with TSA or VPA may lead to cell death, cell viability was evaluated by measuring cellular metabolic activity during treatment using the resazurin reagent. As shown in Figure 2A, after cells were treated with different concentrations of TSA (left panel) or VPA (right panel) for 3, 6 or 24 h, cellular metabolic activity did not change significantly, revealing that HDAC inhibitors do not affect PBMC viability. In this sense, PBMC treatment with 4 mM VPA for 24 h induces a slight but non-significant increase in Annexin V positivity (7% vs. 5%), and the lower concentrations of 2 and 1 mM did not affect the number of cells positive for the apoptosis marker (Figure 2B), suggesting that changes in cytokine expression occur independently of cell death.

### 3.3. VPA Treatment of MDMs Infected with DENV-2 Induces a Significant Decrease in Virus-Induced Inflammatory Cytokine Production

We previously reported that CD14+ cells are a PBMC subpopulation susceptible to DENV infection in vitro and that they are responsible for the production of inflammatory cytokines during infection [17,18]. Considering that VPA induced a decrease in the production of TNF-alpha and IL-6 in DENV-2-infected PBMCs, we evaluated the effect of this drug on a primary culture of human monocyte-derived macrophages treated 3 h before or after infection with DENV-2.

**Figure 3:**
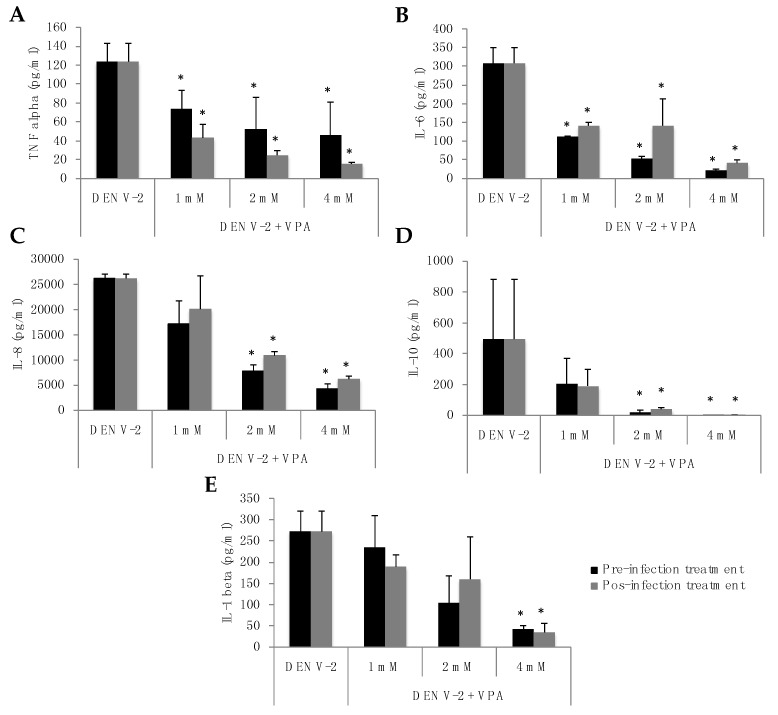
VPA treatment of monocyte-derived macrophages (MDMs) infected with DENV2 induced a significant decrease in inflammatory cytokine production. MDMs were infected with DENV-2 at a multiplicity of infection (MOI) of 1 and treated with VPA (1, 2, or 4 mM) three hours before or after infection. At 24 h post-infection, the supernatants were used to quantify the production of TNF-alpha (**A**); IL-6 (**B**); IL-8 (**C**); IL-10 (**D**) and IL-1beta (**E**). Error bars represent the mean ± SEM of three independent experiments. * *p* values were calculated using the non-parametric two-tailed Mann-Whitney test. A *p* value < 0.05 was considered significant.

MDMs infected with DENV-2 secreted the cytokines IL-8, IL-1b, IL-6, TNF-alpha and IL-10, but IL-12 was not detected under any experimental conditions. Strikingly, VPA treatment had a differential and concentration-dependent effect on cytokine production. For TNF-alpha and IL-6, a significant dose-response reduction was observed under all VPA treatment conditions (Figure 3A,B); for instance, when the cells were treated with 4 mM VPA before infection, the levels of TNF-alpha and IL-6 decreased from 123.7 pg/mL and 308.3 pg/mL to 45.8 pg/mL and 22.2 pg/mL, respectively (reductions of 63% and 92.8%, respectively). Similarly, when 4 mM VPA was applied after infection, the levels of these cytokines decreased to 15.7 pg/mL and 41.4 pg/mL, respectively (reductions of 87.3% and 86.6%, respectively).

For IL-8 and IL-10, significant reductions were observed following treatment with VPA at 2 and 4 mM. Again, the effect was greater when VPA was used at 4 mM (Figure 3C,D). VPA pretreatment at 4 mM induced a decrease of IL-8 and IL-10 mediators from 26,216.8 pg/mL and 495.2 pg/mL to 4221.5 pg/mL and 0.6 pg/mL, respectively (reductions of 83.9% and 99.9%, respectively). The same finding was observed following VPA 4 mM post-treatment of MDMs; the levels of these cytokines decreased to 6196 pg/mL and 1.6 pg/mL, respectively (reductions of 76.4% and 99.7%, respectively).

Finally, IL-1b exhibited a significant reduction in concentration when the cells were treated with VPA at 4 mM (Figure 3E). Thus, treatment of MDM cells with 4 mM VPA for 3 h before infection reduced the level of this cytokine from 272 pg/mL to 41.4 pg/mL (an 84.8% reduction). Similarly, when treatment with VPA was applied 3 h after infection, the level decreased to 34.9 pg/mL (a reduction of 87.2%). Altogether, these data suggest that VPA treatment may induce a strong reduction in the DENV-induced inflammatory cytokine production.

## 4. Discussion

The evidence presented here supports the hypothesis that the inhibition of the activity of HDAC enzymes in DENV-infected human cells significantly reduces the production of the primary cytokines involved in the inflammatory process induced by viral infection. Because these molecules play known roles in disease immunopathogenesis following DENV infection, the modulation or control of their production is an important target for managing this illness, which currently lacks specific pharmacological treatments.

The anti-inflammatory effect of HDAC inhibitors is not a new discovery; this phenomenon had previously been observed in other studies using different models of inflammatory disease [6,19,20]. However, the results presented in this paper describe this effect for the first time using an in vitro infection model with DENV, opening an approach for the development of pharmacological treatments using HDAC inhibitors against DENV-induced disease. In this sense, the first candidate for inclusion in future clinical trials could be valproic acid, a well-characterized drug that has been credited with the ability to inhibit HDAC activity and has been used in the study of therapeutic alternatives for the treatment of inflammatory diseases [21,22,23].

It is important to consider that HDAC inhibitors have many effects on cellular function. The alteration of post-translational protein modifications may induce important biochemical changes in cell survival processes, for instance. In this sense, HDAC inhibitors have been described as antineoplastic agents that promote the death of tumor cells by stimulating the expression of pro-apoptotic proteins, however, this effect is poorly defined in primary cell cultures [24,25,26]; in our study, neither TSA nor VPA induce significant changes in cell viability when they are used in unfractionated PBMCs.

Furthermore, the molecular basis of the effect caused by HDAC inhibitors on the innate immune response is not fully understood [27]. It has been reported, for example, that the inhibition of HDAC activity by TSA decreases the expression of IFN-beta and promotes the replication of Sendai virus in 2fTGH cells; fortunately, it was found that this negative regulation is induced primarily via inhibition of HDAC6 (an HDAC class IIb member) [28], leaving open the possibility that VPA, a selective inhibitor of class I and IIa HDAC enzymes, does not have this effect.

Finally, one important finding in this study was the differential effect of VPA in the production of inflammatory cytokines following DENV infection. Only the highest concentration of VPA resulted in a significant reduction in the production of all cytokines analyzed. This is likely due to the regulation of gene expression by histone acetylation, a complex process whose final effect depends on the site of acetylation and the number of histones acetylated [8,29]. In addition, it is necessary to consider that histones are not the only proteins that undergo this type of post-translational modification [30]. For instance, the acetylation of certain lysines in the p50 and p65 subunits of NF-kB modifies the transcriptional activity of this protein, thereby altering its ability to induce the expression of inflammatory genes [31].

In conclusion, our results clearly support the assumption that valproic acid, an inexpensive and commercially available drug, could have a significant pharmacological effect during the critical phase of dengue virus infection. The discovery of a therapeutic able to modulate the cytokine storm associated with DENV disease and prevent progression to severe illness would represent a significant advance with enormous benefits for both patients and health systems in countries where dengue is endemic.

## Supplementary Materials

The following are available online at http://www.mdpi.com/2079-9721/6/3/59/s1, Figure S1: Morphological development of human monocyte-derived macrophages (MDMs).

## References

[1] Shepard D.S., Undurraga E.A., Halasa Y.A., Stanaway J.D. (2016). The global economic burden of dengue: A systematic analysis. Lancet Infect. Dis..

[2] Guzman M.G., Harris E. (2015). Dengue. Lancet.

[3] Guzman M.G., Gubler D.J., Izquierdo A., Martinez E., Halstead S.B. (2016). Dengue infection. Nat. Rev. Dis. Prim..

[4] Flipse J., Smit J.M. (2015). The complexity of a dengue vaccine: A review of the human antibody response. PLoS Negl. Trop. Dis..

[5] Guy B., Jackson N. (2016). Dengue vaccine: Hypotheses to understand CYD-TDV-induced protection. Nat. Rev. Microbiol..

[6] Das Gupta K., Shakespear M.R., Iyer A., Fairlie D.P., Sweet M.J. (2016). Histone deacetylases in monocyte/macrophage development, activation and metabolism: Refining hdac targets for inflammatory and infectious diseases. Clin. Transl. Immunol..

[7] Roger T., Lugrin J., Le Roy D., Goy G., Mombelli M., Koessler T., Ding X.C., Chanson A.L., Reymond M.K., Miconnet I. (2011). Histone deacetylase inhibitors impair innate immune responses to toll-like receptor agonists and to infection. Blood.

[8] Hull E.E., Montgomery M.R., Leyva K.J. (2016). Hdac inhibitors as epigenetic regulators of the immune system: Impacts on cancer therapy and inflammatory diseases. BioMed Res. Int..

[9] Huber L.C., Brock M., Hemmatazad H., Giger O.T., Moritz F., Trenkmann M., Distler J.H., Gay R.E., Kolling C., Moch H. (2007). Histone deacetylase/acetylase activity in total synovial tissue derived from rheumatoid arthritis and osteoarthritis patients. Arthritis Rheum..

[10] Gunawardhana L.P., Gibson P.G., Simpson J.L., Powell H., Baines K.J. (2014). Activity and expression of histone acetylases and deacetylases in inflammatory phenotypes of asthma. Clin. Exp. Allergy.

[11] Owens M.J., Nemeroff C.B. (2003). Pharmacology of valproate. Psychopharmacol. Bull..

[12] Gottlicher M., Minucci S., Zhu P., Kramer O.H., Schimpf A., Giavara S., Sleeman J.P., Lo Coco F., Nervi C., Pelicci P.G. (2001). Valproic acid defines a novel class of hdac inhibitors inducing differentiation of transformed cells. EMBO J..

[13] Grabiec A.M., Tak P.P., Reedquist K.A. (2011). Function of histone deacetylase inhibitors in inflammation. Crit. Rev. Immunol..

[14] Velandia-Romero M.L., Acosta-Losada O., Castellanos J.E. (2012). In vivo infection by a neuroinvasive neurovirulent dengue virus. J. Neurovirol..

[15] Chen Y.C., Wang S.Y. (2002). Activation of terminally differentiated human monocytes/macrophages by dengue virus: Productive infection, hierarchical production of innate cytokines and chemokines, and the synergistic effect of lipopolysaccharide. J. Virol..

[16] Pfaffl M.W. (2001). A new mathematical model for relative quantification in real-time RT-PCR. Nucleic Acids Res..

[17] Delgado F.G., Pérez-Acosta M., Castellanos J.E. (2014). Descripción de un modelo de infección in vitro con virus dengue empleando células mononucleares humanas de sangre periférica. Iatreia.

[18] Pérez-Acosta M., Delgado F.G., Castellanos J.E. (2013). Soluble ST2 does not regulate TNF- and IL-6 production in dengue virus-infected human monocytes. ISRN Trop. Med..

[19] Castelo-Branco G., Stridh P., Guerreiro-Cacais A.O., Adzemovic M.Z., Falcao A.M., Marta M., Berglund R., Gillett A., Hamza K.H., Lassmann H. (2014). Acute treatment with valproic acid and l-thyroxine ameliorates clinical signs of experimental autoimmune encephalomyelitis and prevents brain pathology in da rats. Neurobiol. Dis..

[20] Cantley M.D., Fairlie D.P., Bartold P.M., Marino V., Gupta P.K., Haynes D.R. (2015). Inhibiting histone deacetylase 1 suppresses both inflammation and bone loss in arthritis. Rheumatol. Oxf..

[21] Zhang Z., Zhang Z.Y., Fauser U., Schluesener H.J. (2008). Valproic acid attenuates inflammation in experimental autoimmune neuritis. Cell. Mol. Life Sci..

[22] Cetinkaya M., Cansev M., Cekmez F., Tayman C., Canpolat F.E., Kafa I.M., Yaylagul E.O., Kramer B.W., Sarici S.U. (2015). Protective effects of valproic acid, a histone deacetylase inhibitor, against hyperoxic lung injury in a neonatal rat model. PLoS ONE.

[23] Costalonga E.C., Silva F.M., Noronha I.L. (2016). Valproic acid prevents renal dysfunction and inflammation in the ischemia-reperfusion injury model. BioMed Res. Int..

[24] Armeanu S., Pathil A., Venturelli S., Mascagni P., Weiss T.S., Gottlicher M., Gregor M., Lauer U.M., Bitzer M. (2005). Apoptosis on hepatoma cells but not on primary hepatocytes by histone deacetylase inhibitors valproate and itf2357. J. Hepatol..

[25] Kalanxhi E., Risberg K., Barua I.S., Dueland S., Waagene S., Andersen S.N., Pettersen S.J., Lindvall J.M., Redalen K.R., Flatmark K. (2017). Induction of apoptosis in intestinal toxicity to a histone deacetylase inhibitor in a phase i study with pelvic radiotherapy. Cancer Res. Treat..

[26] Sweet M.J., Shakespear M.R., Kamal N.A., Fairlie D.P. (2012). Hdac inhibitors: Modulating leukocyte differentiation, survival, proliferation and inflammation. Immunol. Cell Biol..

[27] Grabiec A.M., Potempa J. (2018). Epigenetic regulation in bacterial infections: Targeting histone deacetylases. Crit. Rev. Microbiol..

[28] Nusinzon I., Horvath C.M. (2006). Positive and negative regulation of the innate antiviral response and beta interferon gene expression by deacetylation. Mo. Cell. Biol..

[29] Strahl B.D., Allis C.D. (2000). The language of covalent histone modifications. Nature.

[30] Kori Y., Sidoli S., Yuan Z.F., Lund P.J., Zhao X., Garcia B.A. (2017). Proteome-wide acetylation dynamics in human cells. Sci. Rep..

[31] Spange S., Wagner T., Heinzel T., Kramer O.H. (2009). Acetylation of non-histone proteins modulates cellular signalling at multiple levels. Int. J. Biochem. Cell Biol..

