# Supplementary material for: Valproic Acid Downregulates Cytokine Expression in Human Macrophages Infected with Dengue Virus
Source: Diseases. 2018 Jul 6;6(3):59. doi: 10.3390/diseases6030059 (PMC6165057; doi:10.3390/diseases6030059)
Supplement: Supplementary file 1 [file diseases-06-00059-s001.pdf]

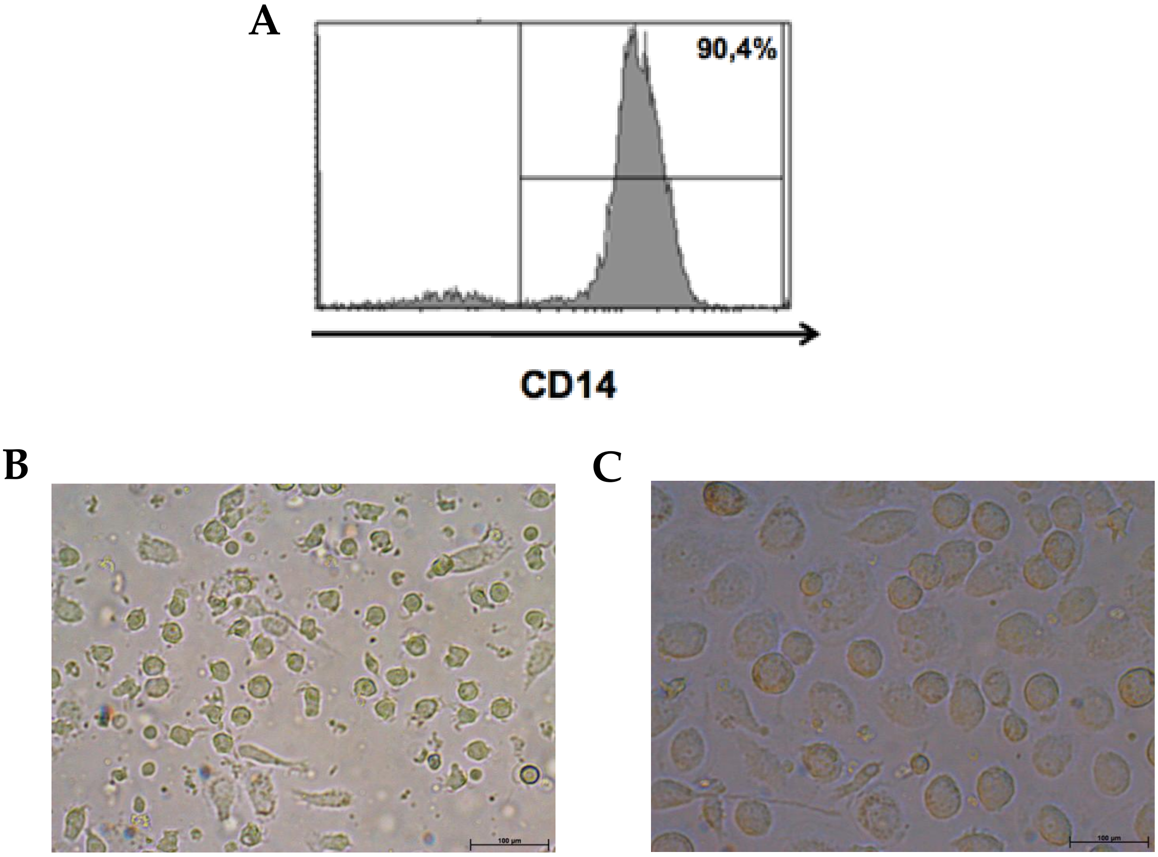

Figure S1. Morphological development of human monocyte-derived macrophages (MDMs). Freshly isolated monocytes obtained from PBMCs by positive selection were stained with an anti-CD14 antibody (A). Cell morphology of monocytes cultured for 1 day (B) and after 7 days of differentiation (C) is shown. Bar correspond to 100 μm.
